# Preoperative Predictors of Exercise-Induced Hypoxemia Following Lung Cancer Surgery

**DOI:** 10.7759/cureus.86813

**Published:** 2025-06-26

**Authors:** Nanako Asaji, Takashi Nakagama, Hirofumi Uehara

**Affiliations:** 1 Department of Rehabilitation, Hakodate Goryokaku Hospital, Hakodate, JPN; 2 Department of Thoracic Surgery, Hakodate Goryokaku Hospital, Hakodate, JPN

**Keywords:** exercise-induced hypoxemia (eih), lung cancer, lung resection, physical function, sarcopenia

## Abstract

Objective: This study aimed to identify preoperative predictors of postoperative exercise-induced hypoxemia (EIH) in patients undergoing lung cancer surgery, with a particular focus on physical function and sarcopenia.

Methods: This single-center, retrospective study included 200 patients who underwent lung resection and perioperative rehabilitation for primary lung cancer between January 2020 and December 2021. The exclusion criteria were incomplete clinical data, no preoperative rehabilitation, and in-hospital death. Postoperative EIH was defined as a ≥4% drop in peripheral oxygen saturation (SpO_2_) during the six-minute walk test (6MWT) prior to discharge. Background characteristics, pulmonary function, physical function, and surgical factors were compared. Multivariate logistic regression was used to examine associations between preoperative variables and postoperative EIH. Additionally, receiver operating characteristic (ROC) curve analysis was conducted to assess the predictive performance of variables identified as significant predictors in the multivariate analysis.

Results: After applying the exclusion criteria, a total of 157 patients were ultimately included and divided into a non-EIH group (n=124) and an EIH group (n=33). Significant differences were observed between the two groups in history of interstitial lung disease, Brinkmann’s index, first second of forced expiratory volume (FEV₁), FEV₁/forced vital capacity (FVC), percentage diffusing capacity of the lungs for carbon monoxide (%DLCO), and resting SpO₂ (p < 0.05 for all). In contrast, no significant differences were found in preoperative physical function or the presence of sarcopenia. In multivariate analysis, higher Brinkmann's index/100 (odds ratio (OR): 1.110; 95% confidence interval (CI): 1.030-1.170; p = 0.003) and lower %DLCO (OR: 0.976; 95%CI: 0.960-0.992; p = 0.004) were associated with a higher likelihood of postoperative EIH. ROC analysis showed that Brinkmann's index/100 had an area under the curve (AUC) of 0.702 and %DLco an AUC of 0.671, with cutoff values of 7.50 and 103.8%, respectively.

Conclusion: Although preoperative physical function and sarcopenia were not significantly associated with the development of postoperative EIH, both Brinkmann's index and %DLCO were associated with increased risk, suggesting their potential utility as preoperative risk markers. These findings may help inform perioperative risk stratification and guide rehabilitation planning for patients undergoing lung cancer surgery. Limitations include the single-center retrospective design, small sample size, possible unmeasured confounders, undetermined optimal cutoff value, and lack of outcome validation related to postoperative EIH.

## Introduction

Advances in less invasive surgical techniques and perioperative management have led to a decline in complication rates following lung cancer surgery [1]. As a result, surgical indications have expanded to include high-risk patients, such as those with reduced physical reserve, older age, impaired respiratory function, or multiple comorbidities [1-3].

In some of these patients, postoperative exercise-induced hypoxemia (EIH) can occur and may necessitate long-term oxygen therapy. Indeed, previous studies have reported that 3.7-15.3% of patients undergoing pulmonary resection for primary lung cancer require continued oxygen therapy [2-4]. Factors associated with postoperative EIH, or the initiation of oxygen therapy, include surgical method, preoperative EIH, pulmonary function, and body mass index (BMI) [2,5,6].

Moreover, recent research highlights the clinical importance of assessing physical function and sarcopenia in predicting postoperative complications and long-term outcomes [7,8]. Sarcopenia, characterized by loss of skeletal muscle mass and strength, is one of the key features of skeletal muscle dysfunction and has been associated with reduced exercise capacity, prolonged hospitalization, and increased postoperative complications. Physiologically, skeletal muscle dysfunction, particularly involving respiratory and peripheral muscles, may impair ventilatory reserve and oxygen utilization during exertion, potentially predisposing patients to EIH [9]. However, no studies to date have examined the association between physical function or sarcopenia and postoperative EIH.

Given that high-risk patients often present with reduced physical function and muscle mass, we hypothesized that postoperative EIH may also be associated with these factors. This study aimed to identify preoperative predictors of EIH after lung cancer surgery, with a particular focus on physical function and sarcopenia.

## Materials and methods

This was a single-center, retrospective study conducted at the Hakodate Goryokaku Hospital, Hakodate, Hokkaido, Japan. This study was conducted in compliance with the Declaration of Helsinki and the Ethical Guidelines for Medical Research Involving Human Subjects and was approved by the Hakodate Goryokaku Hospital Ethics Committee on May 15, 2020 (approval number: 2020-008). Although the data collection started in January 2020, the use of anonymized, pre-existing data was approved under the retrospective design.

Participants

A total of 200 consecutive patients who underwent lung resection for primary lung cancer and perioperative rehabilitation at our institution from January 2020 to December 2021 were included in the study. The exclusion criteria were incomplete clinical data, no preoperative rehabilitation, and in-hospital death.

Postoperative rehabilitation was conducted for all patients according to a standardized protocol, which included respiratory training such as deep breathing and incentive spirometry, and early mobilization starting from the day after surgery. There was minimal individual variation in the intensity of rehabilitation.

Definition of EIH

EIH was defined as a ≥4% decrease in SpO₂ measured before and after the six-minute walk test (6MWT) conducted prior to hospital discharge, indicating exercise-induced desaturation, in accordance with previous studies [10,11]. This definition is commonly used to capture clinically relevant desaturation during exertion, although the absolute SpO₂ value after walking was not necessarily below 90% in all cases. Thus, the presence of EIH in this study reflects impaired pulmonary gas exchange capacity during exercise.

Variables of interest

The variables of interest included age, sex, Brinkmann’s index, medical history, including chronic obstructive pulmonary disease (COPD), interstitial lung disease (ILD), and cardiovascular disease (CVD), and clinical stage. Preoperative pulmonary function tests included vital capacity (VC), %VC, forced expiratory volume in one second (FEV_1_), FEV_1_/forced vital capacity (FVC), and percentage diffusing capacity of the lungs for carbon monoxide (%DLCO). Surgery-related variables included surgical approach, extent of resection, surgical duration, and intraoperative bleeding. Postoperative variables included days to mobilization, six-minute walk distance (6MD) and SpO₂ at discharge, complications, duration of pleural drainage, and length of hospital stay.

Assessment of physical function

A physical therapist preoperatively evaluated hand grip and knee extension strength and skeletal mass index (SMI) and performed the short physical performance battery (SPPB) [12] and 6MWT. The presence of sarcopenia was determined based on the diagnostic criteria established by the Asian Working Group for Sarcopenia in 2019 (AWGS2019) [13]. Hand grip strength, SMI, and SPPB score were classified as normal/decreased based on AWGS criteria (decreased: hand grip strength of <28 and <18 kg for men and women, SMI of <7.0 and <5.7 kg/m^2^ for men and women, and SPPB ≤9).

Hand grip strength was measured twice for both the dominant and nondominant hands (i.e., two times per hand, totaling four measurements), using a JAMAR Hydraulic Hand Dynamometer (SH-5001; SAKAI Medical Co., Ltd, Tokyo, Japan), with the higher values being recorded. For knee extension strength, the maximum isometric extension and contraction were measured at 90° flexion using a hand-held dynamometer (μ-tas F-1, Anima Corporation, Tokyo, Japan). SMI was calculated by dividing the amount of limb skeletal muscle measured by a bioimpedance body composition analyzer (MC-780A, TANITA Corporation, Tokyo, Japan) by the square of the patient’s height. SPPB consists of three tests: a balance test, a four-minute walk test, and the five-times sit-to-stand test. The total score for the SPPB ranged from 0 to 12. The 6MWT was performed according to ATS guidelines [14]. The 6MD, SpO2 changes before and after 6MWT (ΔSpO2), and the presence of EIH were recorded. SpO2 measured using a pulse oximeter (pulsefit BO-750, Nihon Seimitsu Sokki Co., Ltd, Gunma, Japan). The median timing of the 6MWT before discharge was 7.0 days after surgery (interquartile range (IQR): 6.0-8.0).

At our institution, the criteria for discharge include adequate pain control, the ability to tolerate oral intake, and the recovery of activities of daily living (ADL) to the preoperative level. In principle, 6MWT was performed on the day before discharge once these criteria were met and discharge was planned.

Statistical analysis

Continuous variables were presented as mean ± standard deviation (SD) when normally distributed and as median (IQR) when nonnormally distributed. Categorical variables were presented as numbers. The Shapiro-Wilk test was used to determine the normality of data distribution, whereas the unpaired t-test, Mann-Whitney U test, and chi-square test were used to compare patient background factors between the two groups, with p values of <0.05 indicating statistical significance. Multivariate logistic regression analysis was performed using the forced entry method to evaluate associations between selected preoperative variables and postoperative EIH.

Variables were selected based on prior literature and clinical relevance. We chose %DLCO and the Brinkmann's index due to their established roles as indicators of pulmonary gas exchange and smoking exposure, respectively. To improve interpretability and avoid very small odds ratios (ORs), Brinkmann's index was divided by 100 (denoted as Brinkmann's index/100) prior to logistic regression analysis. Sarcopenia and the extent of surgical resection were included based on prior studies showing their associations with postoperative pulmonary complications, including hypoxemia. Sarcopenia reflects reduced physical and respiratory reserve, contributing to impaired recovery and increased respiratory vulnerability [8]. Extensive resection, such as lobectomy, has been reported to increase the risk of postoperative complications, including respiratory impairment, due to the greater loss of lung tissue and consequent reduction in ventilatory capacity and gas exchange efficiency [15].

Univariable logistic regression analyses were also conducted for these selected variables to assess their individual associations with EIH. Multicollinearity among the independent variables was assessed using the variance inflation factor (VIF), and all VIF values were approximately 1, indicating no significant multicollinearity. ORs and 95% confidence intervals (CIs) were calculated to estimate the strength. The goodness of fit of the final model was evaluated using the Hosmer-Lemeshow test, with p > 0.05 indicating an acceptable fit. Additionally, receiver operating characteristic (ROC) curve analysis was conducted to assess the predictive performance of variables identified as significant predictors in the multivariate analysis. Optimal cutoff values were determined using the Youden index. All statistical analyses were performed using EZR (Easy R), version 1.68 (Saitama Medical Center, Jichi Medical University, Saitama, Japan), a graphical user interface for R [16].

## Results

**Figure 1:**
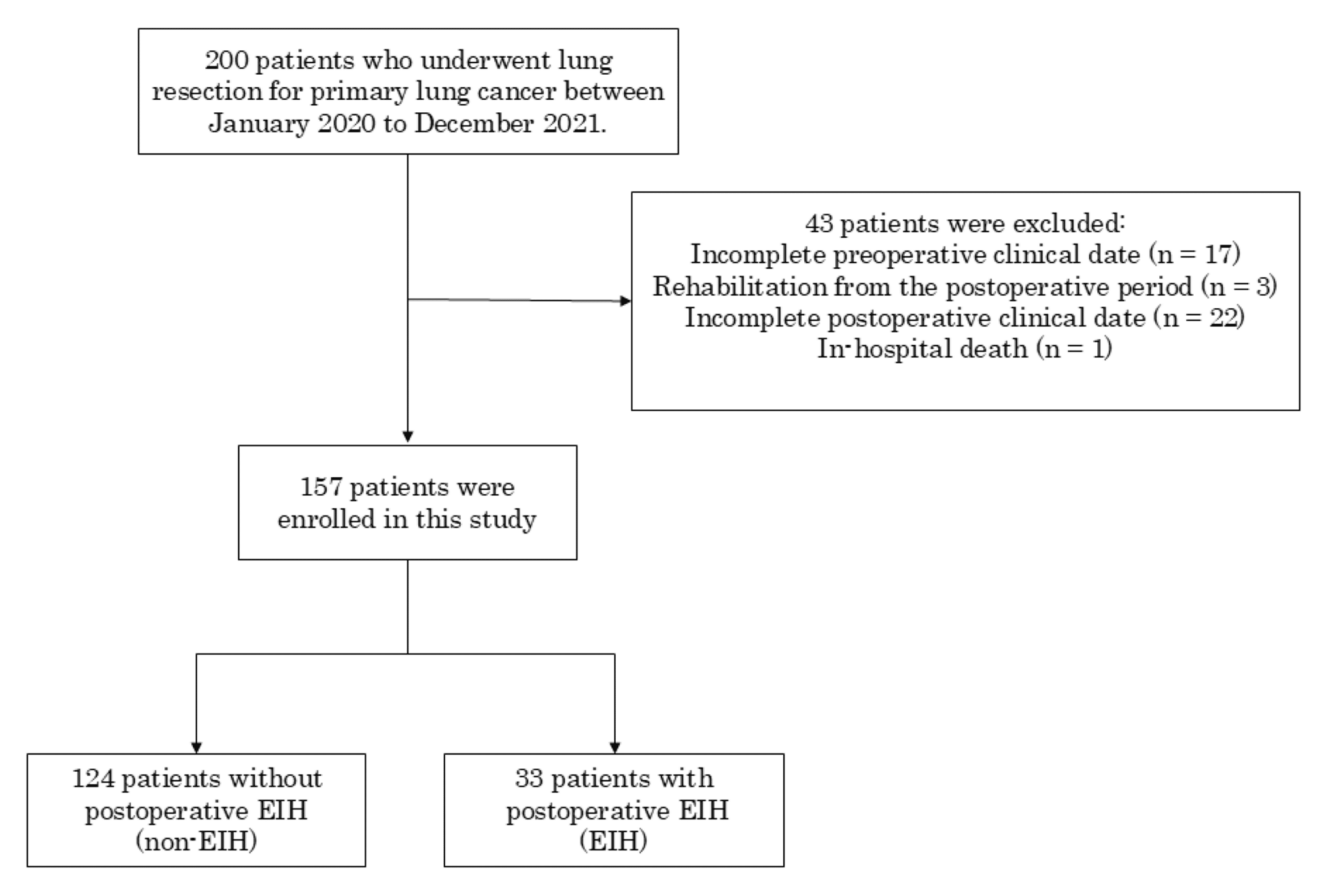
Flow diagram showing participant selection EIH, exercise-induced hypoxemia

After applying the exclusion criteria, a total of 157 patients were ultimately included and divided into two groups according to the presence or absence of postoperative EIH. The study cohort comprised 124 patients in the non-EIH group and 33 in the EIH group (Figure 1).

Significant differences regarding history of ILD, Brinkmann’s index, FEV1, FEV1/FVC, %DLCO, and Pre-6MWT SpO2 were observed between the two groups (p < 0.05). However, no significant differences in surgery-related factors, muscle strength, SMI, SPPB score, presence of sarcopenia, 6MD, Post-6MWT SpO2, ΔSpO2, and preoperative EIH were observed between the two groups (Tables 1, 2).

**Table 1:** Characteristics between non-EIH and EIH Values are presented as median (IQR), mean±SD, or n (%). Statistical tests used: Mann–Whitney U test or unpaired t-test for continuous variables; chi-square test or Fisher’s exact test for categorical variables. Test statistics (U, t or χ² values) are shown; “N/A” indicates not applicable (e.g., Fisher’s exact test). EIH, exercise-induced hypoxemia; BMI, body mass index; COPD, chronic obstructive pulmonary disease; ILD, interstitial lung disease; VATS, video-assisted thoracic surgery; IQR, interquartile range

	Non-EIH (n=124)	EIH (n=33)	Test statistic	p-value
Age (years), median (IQR)	72.5 (67.0-77.0)	73.0 (68.0-77.0)	U=1985.5	0.796
Sex (male/female), n	75/49	21/12	χ2=0.016	0.897
BMI (kg/m^2^), median (IQR)	23.6 (21.5-25.8)	23.3 (20.6-26.4)	U=2156.0	0.637
Comorbidity, n (%)				
COPD	8 (6.4%)	4 (12.1%)	N/A	0.279
ILD	1 (0.8%)	3 (9.0%)	N/A	0.029
Cardiovascular disease	20 (16.1%)	2 (6.0%)	N/A	0.168
Brinkmann’s index, median (IQR)	400 (0-905)	860 (495-1480)	U=1346.0	0.002
Clinical stage (0/1/2/3/4), n	2/104/14/4/0	1/26/6/0/0	N/A	0.482
Surgical approach, n (%)				
VATS	118 (95.1%)	30 (90.9%)		
Open	6 (4.8%)	3 (9.0%)	N/A	0.398
Extent of resection, n (%)				
Partial resection	15 (12.0%)	7 (21.2%)		
Segmentectomy	4 (3.2%)	3 (9.0%)		
Lobectomy	105 (84.6%)	23 (69.6%)	N/A	0.103
Surgical duration (minutes), mean±SD	179.5±62.2	181.2±63.2	t=-0.142	0.887
Intraoperative bleeding (ml), median (IQR)	20.0 (10.0-33.2)	12.0 (9.0-30.0)	U=2283.0	0.307

**Table 2:** Preoperative respiratory and physical functions between non-EIH and EIH Values are presented as median (IQR), mean±SD, or n (%). Statistical tests used: Mann–Whitney U test or unpaired t-test for continuous variables; Fisher’s exact test for categorical variables. Test statistics (U, t values) are shown; “N/A” indicates not applicable (e.g., Fisher’s exact test). EIH, exercise induced hypoxemia; VC, vital capacity; FEV_1_, forced expiratory volume in one second; FVC, forced volume capacity;  %DLco, percent predicted diffusing capacity for carbon monoxide; SMI, skeletal muscle index; SPPB, short physical performance battery; 6MD, 6-minute walk distance; 6MWT, 6-minute walk test; IQR, interquartile range

	non-EIH (n=124)	EIH (n=33)	Test statistic	p-value
Respiratory function				
VC (L), median (IQR)	3.1 (2.6-3.6)	3.0 (2.6-3.4)	U=2119.5	0.753
%VC (%), mean±SD	102.7±16.0	102.0±16.3	t=0.221	0.825
FEV1 (L), median (IQR)	2.3 (1.9-2.6)	2.0 (1.6-2.4)	U=2565.5	0.025
FEV1/FVC (%), median (IQR)	76.1 (71.3-81.0)	68.7 (61.6-78.5)	U=2505.5	0.048
%DLCO (%), median (IQR)	113.0 (100.2-133.6)	100.1 (81.1-114.2)	U=2873.0	<0.001
Knee extension strength (kgf/kg), mean±SD	0.4±0.1	0.5±0.1	t=-0.396	0.692
Hand grip strength (kg), median (IQR)	30.0 (24.0-35.5)	30.0 (24.0-37.0)	U=2072.0	0.855
SMI (kg/m^2^), mean±SD	7.3±1.1	7.1±1.1	t=0.827	0.409
SPPB, median (IQR)	12.0 (11.0-12.0)	12.0 (12.0-12.0)	U=1676.5	0.053
Sarcopenia, n (%)	8 (6.4%)	1 (3.0%)	N/A	0.686
6MD (m), median (IQR)	462.5 (411.4-507.5)	456.3 (403.0-511.7)	U=2017.0	0.902
Pre-6MWT SpO2 ( %), median (IQR)	98.0 (97.0-98.0)	97.0 (97.0-98.0)	U=2499.5	0.034
Post-6MWT SpO2 (%), median (IQR)	97.0 (96.0-98.0)	97.0 (96.0-98.0)	U=2376.5	0.137
ΔSpO2 (%), median (IQR)	0.0 (0.0-1.0)	1.0 (0.0-1.0)	U=1810.5	0.286
Preoperative EIH, n (%)	3 (2.4%)	2 (6.0%)	N/A	0.283

Among the postoperative outcomes, only post-6MWT SpO₂ and ΔSpO₂ showed statistically significant differences between the non-EIH and EIH groups (p < 0.001). No significant differences were observed in the days to mobilization, postoperative 6MD, incidence of complications, duration of pleural drainage, or length of postoperative hospital stay (Table 3). Only one patient required home oxygen therapy.

**Table 3:** Postoperative course and outcomes between non-EIH and EIH Values are presented as median (IQR) or n (%). Statistical tests used: Mann–Whitney U test for continuous variables; Fisher’s exact test for categorical variables. Test statistics (U values) are shown; “N/A” indicates not applicable (e.g., Fisher’s exact test). EIH, exercise induced hypoxemia; 6MD, 6-minute walk distance; 6MWT, 6-minute walk test; IQR, interquartile range

	non-EIH (n=124)	EIH (n=33)	Test statistic	p-value
Sitting (days), median (IQR)	1.0 (1.0-1.0)	1.0 (1.0-1.0)	U=2062.5	0.617
Standing (days), median (IQR)	1.0 (1.0-1.0)	1.0 (1.0-1.0)	U=2062.5	0.686
Gait (days), median (IQR)	1.0 (1.0-1.0)	1.0 (1.0-1.0)	U=1928.0	0.272
6MD (m), median (IQR)	416.4 (368.5-461.0)	433.0 (350.0-465.0)	U=1960.0	0.713
Pre-6MWT SpO2 (%), median (IQR)	97 (96-98)	97 (95-97)	U=2310.0	0.232
Post-6MWT SpO2 (%), median (IQR)	97 (95.7-97)	91 (89.0-92.0)	U=4024.5	<0.001
ΔSpO2 (%), median (IQR)	0 (0-1)	5 (4-6)	U=0	<0.001
Complications, n, %	12 (9.6%)	6 (18.1%)	N/A	0.216
Duration of pleural drainage (days), median (IQR)	2.0 (2.0-3.0)	2.0 (2.0-3.0)	U=2234.0	0.382
Length of hospital stay (days), median (IQR)	8.0 (6.0-9.0)	8.0 (7.0-10.0)	U=1756.0	0.205

**Figure 2:**
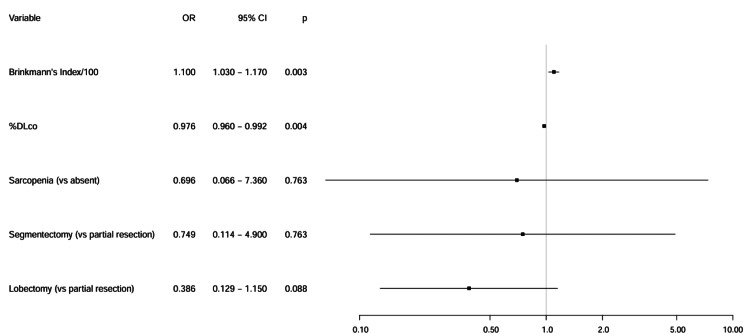
Odds ratio plot of univariate analysis for postoperative EIH EIH, exercise induced hypoxemia; OR, odds ratio; CI, confidence interval; %DLco, percent predicted lung diffusing capacity for carbon monoxide

Univariable logistic regression analyses were performed for the selected preoperative variables. Among these, Brinkmann's index/100 (OR: 1.110, 95% CI: 1.040-1.180, p < 0.001) and %DLco (OR: 0.974, 95% CI: 0.958-0.990, p = 0.001) were significantly associated with postoperative EIH. The presence of sarcopenia and the type of resection were not significantly associated with EIH in univariable analyses (Table 4, Figure 2).

**Table 4:** Univariable logistic regression analysis of selected preoperative variables associated with postoperative EIH EIH, exercise induced hypoxemia; OR, odds ratio; CI, confidence interval; %DLCO, percent predicted lung diffusing capacity for carbon monoxide; Ref, reference group

	OR	95%CI	p-value
Brinkmann’s index/100	1.110	1.040-1.180	<0.001
%DLCO	0.974	0.958-0.990	0.001
Sarcopenia (present)	0.453	0.054-3.760	0.460
Partial resection (Ref)	-	-	-
Segmentectomy	1.610	0.281-9.200	0.594
Lobectomy	0.469	0.172-1.280	0.140

**Figure 3:**
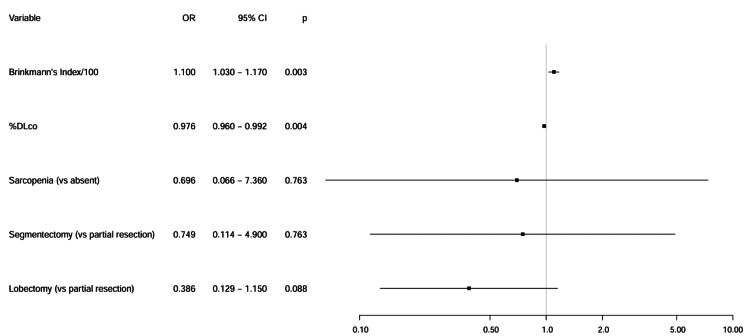
Odds ratio plot of multivariate analysis for postoperative EIH EIH, exercise induced hypoxemia; OR, odds ratio; CI, confidence interval; %DLco, percent predicted lung diffusing capacity for carbon monoxide

Multiple logistic regression analysis resulted in significant regression equations (χ2 = 24.980, p < 0.001), with the Hosmer-Lemeshow analysis showing good fitness (χ2 = 8.214, p = 0.412). Notably, we identified Brinkmann's index/100 (OR: 1.110, 95%CI: 1.030-1.170, p = 0.003) and %DLCO (OR: 0.976, 95%CI: 0.960-0.992, p = 0.004) as factors significantly associated with postoperative EIH (Table 5, Figure 3).

**Table 5:** Risk factors for EIH identified by multivariate logistic regression analysis EIH, exercise induced hypoxemia; OR, odds ratio; CI, confidence interval; %DLCO, percent predicted lung diffusing capacity for carbon monoxide; Ref, reference group

	OR	95%CI	p-value
Brinkmann’s index/100	1.110	1.030-1.170	0.003
%DLCO	0.976	0.960-0.992	0.004
Sarcopenia (present)	0.696	0.065-7.360	0.763
Partial resection (Ref)	-	-	-
Segmentectomy	0.749	0.114-4.900	0.763
Lobectomy	0.386	0.129-1.150	0.088

**Figure 4:**
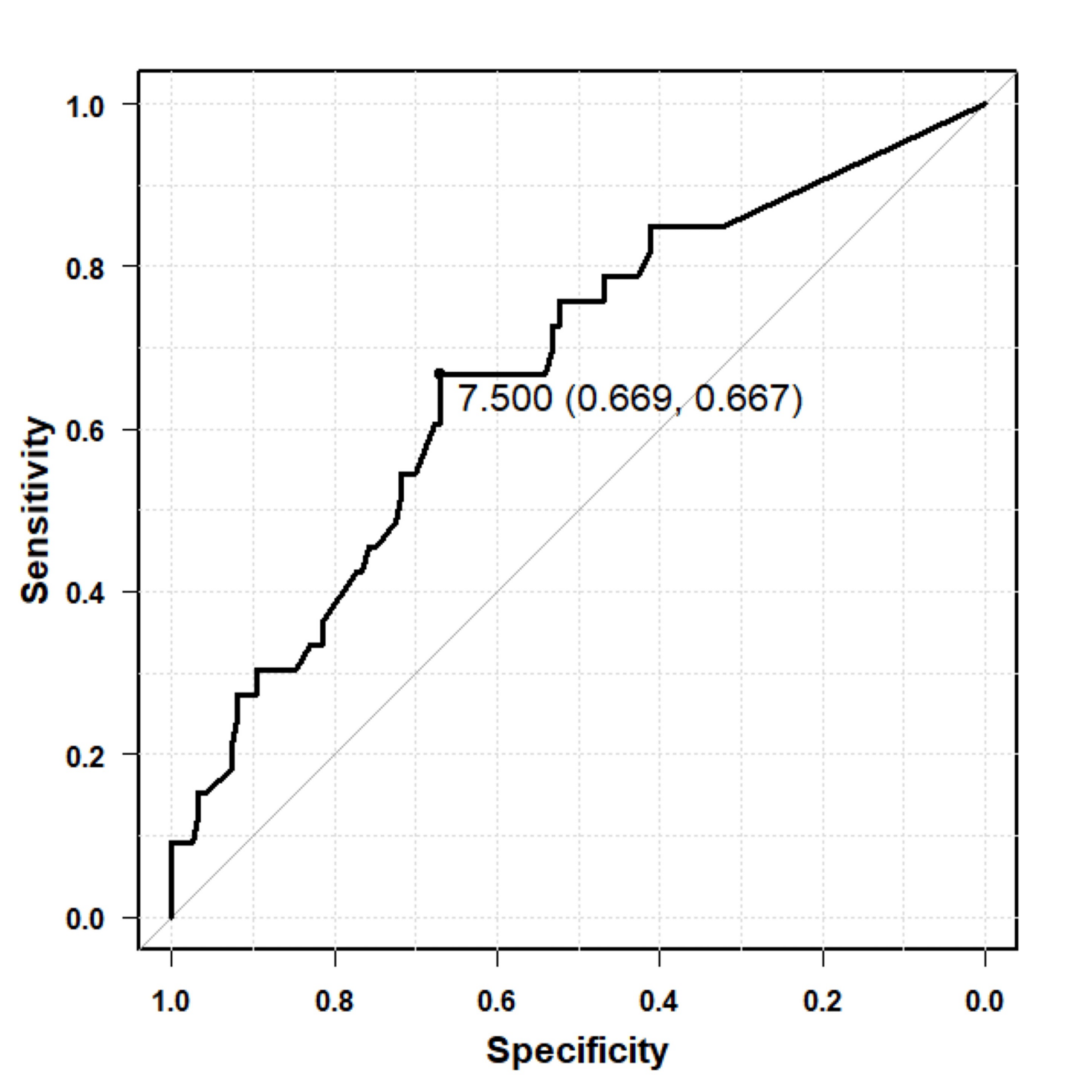
ROC curve for Brinkmann's index (per 100 units) in predicting postoperative EIH The ROC curve displays the discriminative ability of the Brinkmann's index (scaled per 100 units) for predicting postoperative EIH. The AUC was 0.671 (95%CI: 0.566–0.776). The optimal cutoff value determined using the Youden index was 7.50, with a sensitivity of 66.9% and specificity of 66.7%.

**Figure 5:**
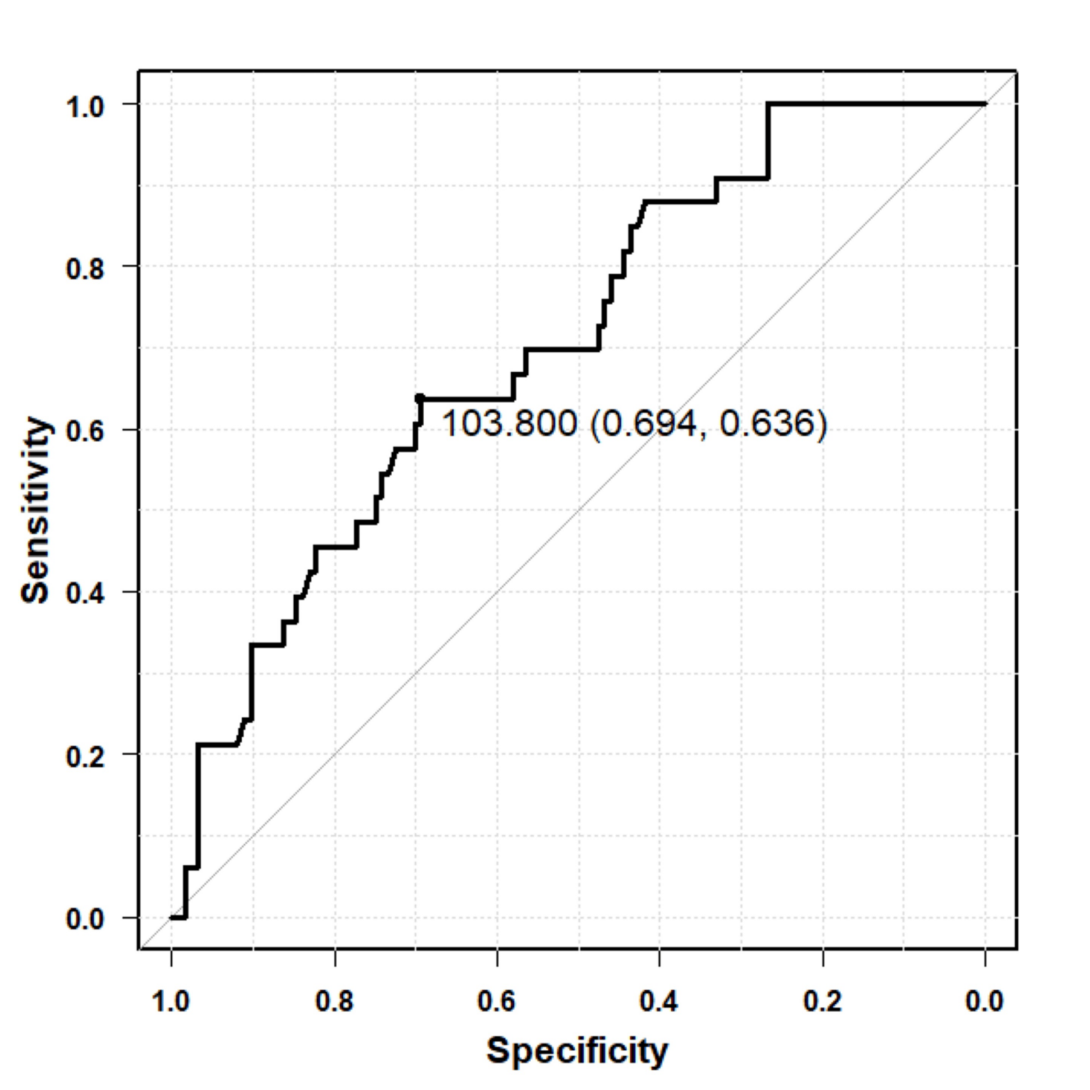
ROC curve for %DLCO in predicting postoperative EIH The ROC curve displays the discriminative ability of preoperative %DLCO for predicting postoperative EIH. The AUC was 0.702 (95%CI: 0.606–0.798). The optimal cutoff value determined using the Youden index was 103.8%, with a sensitivity of 69.4% and specificity of 63.6%. ROC, receiver-operating characteristic; EIH, exercise-induced hypoxemia; AUC, area under the curve; %DLCO, percentage diffusing capacity of the lungs for carbon monoxide

ROC curve analysis demonstrated that Brinkmann's index/100 predicted postoperative EIH with an AUC of 0.671 (95%CI: 0.566-0.776). The optimal cutoff value identified using the Youden index was 7.50, with a sensitivity of 66.9% and specificity of 66.7% (Figure 4). Similarly, %DLCO showed predictive ability for EIH, with an AUC of 0.702 (95%CI: 0.606-0.798). The optimal cutoff value was 103.8%, corresponding to a sensitivity of 69.4% and specificity of 63.6% (Figure 5).

## Discussion

Contrary to our initial hypothesis, preoperative physical function was not significantly associated with the development of postoperative EIH. This finding suggests that baseline functional capacity may not adequately reflect the pulmonary reserve or gas exchange efficiency underlying EIH. One possible explanation is that our study cohort, consisting of patients deemed suitable for surgical intervention, may not have included individuals with sufficiently severe physical dysfunction or sarcopenia to influence the development of postoperative EIH. Nevertheless, the assessment of physical function remains clinically important, as it provides essential information for evaluating patients' overall status, guiding perioperative rehabilitation, and predicting other postoperative outcomes such as physical inactivity and quality of life.

In contrast, our results showed Brinkmann's index/100 and %DLco were significantly associated factors with postoperative EIH. This finding emphasizes the important roles of cumulative smoking exposure and pulmonary diffusion capacity in predicting postoperative EIH, providing valuable insights into potential risk factors for this patient cohort. Pack years have been identified a factor independently associated with diffusion disorder [17], and DLCO is an index of pulmonary gas exchange, focusing on the transfer of oxygen and carbon monoxide between alveoli and capillaries [18], and is influenced by the alveolar membrane area, diffusing gas pressure, and the carrying capacity of the circulating blood volume [19]. Postoperative EIH may result from reduced gas exchange capacity due to the loss of pulmonary capillary beds after lung resection [20].

On the other hand, ROC analysis demonstrated moderate predictive ability for both Brinkmann’s index/100 and %DLCO, with AUCs of 0.671 and 0.702, respectively. The cutoff for Brinkmann’s index/100 was 7.50, with a sensitivity of 66.9% and specificity of 66.7% indicating the threshold of cumulative smoking exposure associated with increased EIH risk. The optimal cutoff value for %DLCO was relatively high at 103.8%, with a sensitivity of 69.4% and specificity of 63.6%, possibly reflecting generally elevated % DLCO levels in this study cohort and the inclusion of mild desaturation cases (SpO₂ ≥90%) in the EIH definition. These findings suggest that while both Brinkmann’s index and %DLCO contribute valuable information for predicting postoperative EIH, their predictive power alone is moderate. Thus, clinical risk stratification should incorporate multiple factors rather than relying on any single marker.

Furthermore, our study showed significant differences in the history of ILD (n=1 vs n=3), Brinkmann’s index (400 vs 860), FEV_1_ (2.3L vs 2.0L), FEV_1_/FVC (76.1% vs 68.7%), %DLCO (113.0% vs 100.1%), and Pre-6MWT SpO₂ (98.0% vs 97.0%) between the non-EIH and EIH groups. EIH in ILD has been significantly associated with diffusion disorder [21,22]. These findings also suggest that EIH may depend on the background lung reserve capacity, particularly gas exchange capacity, and that attention should be paid to background factors associated with diffusion disorders.

Previous studies have shown that preoperative EIH can predict postoperative EIH, including the need for home oxygen therapy, as well as increased risks of complications and mortality [5,23,24]. However, in our study, no significant difference in the prevalence of preoperative EIH was observed between the postoperative non-EIH and EIH groups. This discrepancy may be partly explained by our inclusion of patients whose SpO₂ remained above 90% despite a ≥4% drop; in such cases, the clinical impact of desaturation may have been limited. Furthermore, some patients with preoperative EIH caused by airflow limitation may have experienced postoperative improvement due to bronchodilator use or the lung volume reduction effect of surgical resection. These interventions may have improved ventilation-perfusion matching and thereby mitigated desaturation during postoperative exercise.

Although the guidelines recommend that a pulmonary diffusion capacity test be performed in all patients scheduled for lung resection [25,26], the need for such a test has not been fully recognized in Japan, with only 27% of patients scheduled for lung resection undergoing such a test [27]. DLCO has also been identified as a predictor of complications and long-term survival after lung cancer resection [28-30]. Our results support the significance of measuring DLCO and confirm its validity as a risk marker for postoperative EIH.

There are limited options to improve DLCO in the short term; perioperative rehabilitation may potentially play a vital role and can be provided based on the risk of EIH. Indeed, teaching effective breathing techniques or strategies for conserving energy during daily activities can aid in enhancing respiratory efficiency to prepare for increased dyspnea. Implementing targeted rehabilitation strategies to address EIH may help prevent physical inactivity and improve overall patient outcomes.

Although this was an exploratory, single-center study with a modest sample size, our findings suggest that both Brinkmann's index and %DLCO may have potential utility in identifying patients at increased risk for postoperative EIH. To our knowledge, few studies have simultaneously evaluated these markers in the context of EIH assessed via 6MWT in the early postoperative period. These results may offer preliminary insights into risk stratification for postoperative EIH in real-world rehabilitation settings.

This study has several limitations. First, it was conducted at a single center, and potential biases may have arisen from the retrospective design and scheduled follow-up visits, limiting the generalizability of our findings. Second, the modest sample size, including a limited number of patients with postoperative EIH and patients who met the diagnostic criteria for sarcopenia, may have reduced the statistical power and limited our ability to detect certain associations. In addition, unmeasured confounding factors such as the use of bronchodilators and steroids could have influenced the observed associations. Third, optimal cutoff values for predicting EIH were not determined. Finally, we did not evaluate the association between postoperative EIH and outcomes such as daily physical activity, quality of life, home oxygen therapy, and readmission. Future prospective studies with larger and more diverse cohorts are needed to comprehensively understand the preoperative factors influencing postoperative outcomes in patients with lung cancer undergoing rehabilitation. Although not examined in the present study, future research may benefit from sensitivity or subgroup analyses to further validate these findings.

## Conclusions

Brinkmann's index and %DLCO were significantly associated with postoperative EIH, supporting their use as a practical preoperative marker. Although physical function was not found to be associated with EIH, its assessment remains valuable for perioperative planning. Perioperative rehabilitation based on EIH risk, including breathing techniques and energy conservation strategies, may improve respiratory efficiency, manage dyspnea, and prevent postoperative physical inactivity, thereby enhancing overall patient outcomes. Further research is warranted to explore targeted rehabilitation strategies for patients at risk of EIH.
